# Stent-Assisted Coil Embolization of a True Posterior Communicating Artery Aneurysm: A Case Report

**DOI:** 10.7759/cureus.103267

**Published:** 2026-02-09

**Authors:** Ryo Matsuzaki, Yutaka Fuchinoue, Sho Nitta, Masaaki Nemoto, Nobuo Sugo

**Affiliations:** 1 Department of Neurosurgery, Faculty of Medicine, Toho University, Tokyo, JPN; 2 Department of Neurosurgery, Faculty of Medicine, Toho University, Chiba, JPN

**Keywords:** dual antiplatelet therapy (dapt), fetal type of posterior communicating cerebral arteries, neuroform atlas, stent-assisted coil embolization, true posterior communicating artery aneurysm

## Abstract

True aneurysms arising from the posterior communicating artery (PCoA) are rare. Although most reported cases have been managed microsurgically, advances in low-profile stents and microcatheters have expanded endovascular options while preserving PCoA flow. A 74-year-old woman was incidentally diagnosed with an 8-mm unruptured true PCoA aneurysm on a fetal-type left PCoA. The broad neck and medial projection made microsurgical clipping less suitable. A Neuroform Atlas stent was delivered by advancing a microcatheter from the internal carotid artery into the PCoA. The stent was deployed with both ends anchored within the PCoA, followed by coil embolization using the jailing technique. Complete aneurysm occlusion was achieved with preserved PCoA patency. The patient had no neurological deficits and was discharged on postoperative day 7 with a modified Rankin Scale score of 0. Six-month follow-up imaging confirmed durable occlusion without recanalization. Stent-assisted coiling with a low-profile intracranial stent can be an effective reconstructive option for wide-neck true PCoA aneurysms while maintaining PCoA flow. Long-term imaging follow-up is warranted.

## Introduction

True aneurysms of the posterior communicating artery (PCoA) - defined as aneurysms arising from the PCoA itself distal to its origin from the internal carotid artery (ICA) - are rare, representing approximately 1.3% of all intracranial aneurysms and 6.8% of aneurysms in the PCoA region [1]. Several reports suggest that these lesions have a high propensity for rupture compared with typical ICA-PCoA junctional aneurysms [1]. Historically, most true PCoA aneurysms were managed with microsurgical clipping. However, surgical treatment can be challenging due to the deep operative corridor, short arterial segment, and proximity to perforators and cranial nerves, sometimes necessitating complex skull base approaches [1,2].

In the endovascular era, coil embolization has become an attractive alternative because it avoids brain retraction and direct manipulation of delicate perforators [3,4]. However, wide-neck morphologies remain technically difficult, as coils alone may be unstable or compromise the parent artery. This is particularly critical in fetal-type PCoA configurations where the posterior cerebral artery (PCA) territory depends on ICA inflow, necessitating strategies that preserve PCoA patency [5]. While stent-assisted coiling (SAC) is established for wide-neck aneurysms, its application in true PCoA aneurysms, particularly those with fetal-type anatomy and small vessel caliber (<2 mm), presents unique challenges. This report details the technical nuances of using a low-profile stent (Neuroform Atlas) to preserve a hemodynamically critical PCoA.

## Case presentation

Clinical history and imaging

**Figure 1:**
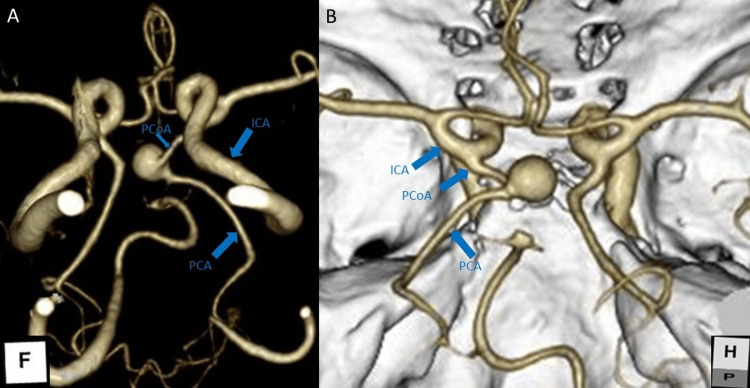
Computed tomography angiography (CTA) of the brain. (A) CTA image showing a saccular aneurysm arising medially from the left PCoA. (B) Fetal-type configuration of the left PCoA is observed, with an absent P1 segment of the PCA. ICA, internal carotid artery; PCoA, posterior communicating artery; PCA, posterior cerebral artery

**Figure 2:**
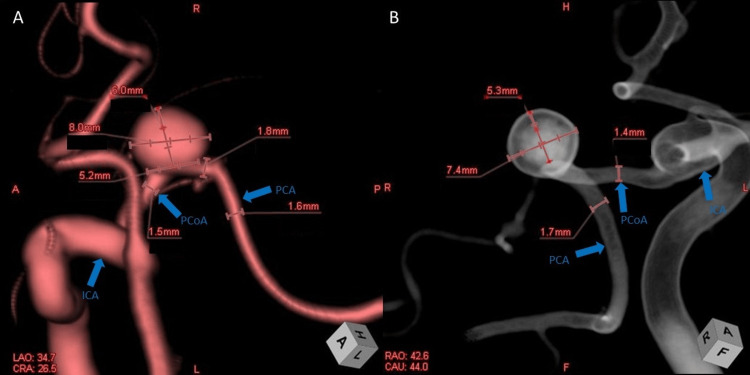
Pre-treatment 3D rotational angiography. (A) Volume-rendered 3D DSA shows a true aneurysm arising from the left PCoA. (B) Translucent-rendered barrel view of the parent artery for evaluating the aneurysm neck. This view enhances visualization of intraluminal relationships and vessel curvature. The aneurysm exhibits a wide neck and medial projection. ICA, internal carotid artery; PCoA, posterior communicating artery; PCA, posterior cerebral artery; DSA, digital subtraction angiography

A 74-year-old woman was referred to our neurosurgery service after an incidental aneurysm was detected on brain imaging performed for intermittent dizziness. She had a history of hypertension but no prior neurologic deficits. On admission, her neurological examination was normal. Computed tomography angiography (CTA) and subsequent digital subtraction angiography (DSA) revealed a saccular aneurysm arising from the medial surface of the left PCoA, distal to its origin from the ICA (Figures 1A, 1B). The aneurysm measured 8 mm (dome width) × 6 mm (height) with a 5.2 mm neck (Figure 2A).

Crucially, the left PCoA exhibited a fetal-type configuration with an angiographically absent P1 segment of the PCA (Figures 1A, 1B). Given this anatomical configuration, the left PCA territory was entirely dependent on ICA inflow through the PCoA. Consequently, an Allcock test (carotid compression test) was not performed, as the risk of inducing ischemia in the PCA territory outweighed the diagnostic benefit. A reconstructive endovascular strategy was planned to secure the aneurysm while preserving PCoA flow.

Pre-procedural management

To minimize thrombotic complications associated with stent placement, dual antiplatelet therapy (aspirin 100 mg and clopidogrel 75 mg daily) was initiated 14 days before the procedure. Platelet function testing was not performed; however, strict medication compliance was confirmed, and the patient had no history of bleeding diathesis.

Endovascular procedure

The intervention was performed under general anesthesia with systemic heparinization to maintain an activated clotting time between 250 and 300 seconds. A 6 French guiding catheter was positioned in the left ICA. The procedure utilized SAC. First, a microcatheter (SL-10; Stryker Neurovascular, Fremont, CA) with a pre-shaped 45-degree tip was carefully navigated into the distal PCoA, past the aneurysm neck. Navigating the acute ICA-PCoA angle was challenging; however, the pre-shaped tip and a flexible microwire allowed for successful distal access without vasospasm. Although individual thalamoperforating arteries were too small to be visualized on DSA, their preservation was a priority.

**Figure 3:**
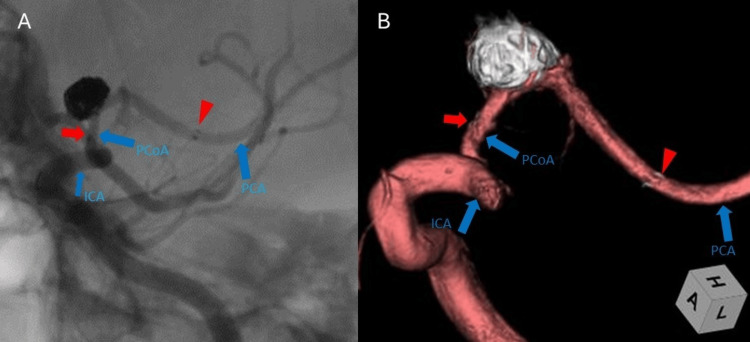
Post-treatment digital subtraction angiography (DSA) of the left ICA. (A) 2D DSA showing near-complete occlusion with a tiny neck remnant after stent-assisted coil embolization. Flow through the parent artery (PCoA) is preserved. The red arrow (→) indicates the proximal end of the stent, and the red arrowhead marks the distal end within the PCoA. (B) 3D rotational angiographic image acquired from left ICA injection. The image is cropped at the level of the ICA bifurcation to improve clarity of the stented area. The proximal and distal ends of the stent are marked by the red arrow and red arrowhead, respectively, as shown in panel (A). ICA, internal carotid artery; PCoA, posterior communicating artery; PCA, posterior cerebral artery

A second microcatheter was navigated into the aneurysm sac. A Neuroform Atlas stent (3 mm × 21 mm; Stryker Neurovascular) was then deployed via the first microcatheter. The stent was positioned entirely within the PCoA, extending from the distal segment back to the proximal segment to bridge the aneurysm neck, with the proximal marker landed just distal to the PCoA origin without protruding into the ICA. The open-cell design of this stent was selected to provide excellent wall apposition in the curved vessel while minimizing metal coverage over potential perforator origins. Following stent deployment, eight detachable platinum coils were inserted through the jailed microcatheter. Post-embolization angiography demonstrated near-complete occlusion with a tiny neck remnant, classified as Raymond-Roy Occlusion Class 2 (residual neck), and full preservation of antegrade flow through the PCoA (Figures 3A, 3B).

Postoperative course

The patient awakened from anesthesia without any new neurological deficits, suggesting that blood flow to the perforating branches and the distal PCA territory was preserved. She was mobilized the following day and discharged home on postoperative day 7 with a modified Rankin Scale score of 0. Dual antiplatelet therapy was continued for three months, followed by indefinite aspirin monotherapy. Follow-up magnetic resonance angiography at six months confirmed durable occlusion of the aneurysm (Raymond-Roy Occlusion Class 1: complete occlusion) with a patent PCoA.

## Discussion

True PCoA aneurysms, arising from the PCoA itself distal to its origin from the internal carotid artery (ICA), are rare lesions and differ from the far more common ICA-PCoA junctional aneurysms in both anatomical context and technical implications for treatment [1]. Because the PCoA is a short vessel with critical perforators and variable contribution to posterior circulation perfusion, therapeutic strategies must balance durable aneurysm exclusion against the risk of compromising parent artery flow and adjacent branches.

Hemodynamic factors are widely considered central to the pathogenesis of true PCoA aneurysms. A fetal-type configuration can create a functional dependence of the PCA territory on ICA inflow through the PCoA. When the P1 segment is absent, as in our case, the clinical consequences of PCoA compromise may be substantial [5]. Accordingly, reconstructive approaches are favored over parent artery occlusion when the PCoA serves as a dominant inflow route.

Microsurgical considerations

Microsurgical clipping has historically been the predominant treatment for true PCoA aneurysms [1]. However, the operative corridor is deep, the accessible arterial segment is short, and the aneurysm is frequently adjacent to perforators and cranial nerves. These considerations are amplified when the aneurysm projects medially, as in our patient, where clip application and proximal control may be challenging without manipulating the oculomotor nerve or perforating arteries [2].

Endovascular options and stent selection for small vessels

Endovascular therapy avoids brain retraction and direct manipulation of perforators. However, wide-neck morphology remains a key limitation for coiling alone. SAC provides scaffolding across the neck and improves coil stability [6]. A critical challenge in true PCoA aneurysms is the small caliber of the parent vessel. In our case, the PCoA diameter was less than 2 mm. Selection of the stent platform is particularly important in this context. Ozaki et al. reported the feasibility of SAC using the Neuroform Atlas stent for wide-neck aneurysms in arteries less than 2 mm in diameter, demonstrating that stent deployment can be performed with acceptable angiographic and clinical outcomes in carefully selected cases [7]. Based on this evidence, we selected the Neuroform Atlas for its low-profile delivery system and open-cell design, which facilitates wall apposition in curved segments and minimizes the metal surface area over perforator origins.

Technical considerations for navigation

The most technically challenging step in this procedure is often microcatheter navigation from the ICA into the PCoA, given the acute branching angle. In our experience, utilizing a microcatheter with a pre-shaped 45-degree tip, rather than a straight tip, significantly facilitated the selection of the PCoA origin. Furthermore, stable support from the guiding catheter in the ICA is essential to prevent the microcatheter from kicking back during distal navigation. Once the PCoA is selected, careful wire handling is required to avoid spasm or dissection in the small-caliber vessel.

Antiplatelet considerations

While ruptured presentations introduce complex dilemmas regarding antiplatelet management, unruptured cases allow for elective preparation. In our patient, dual antiplatelet therapy was initiated 14 days before the procedure to ensure adequate platelet inhibition. This preparation is crucial when placing stents in small, low-flow vessels like the PCoA to prevent acute thrombosis. The absence of ischemic complications in our case supports the safety of this approach when managing unruptured true PCoA aneurysms.

Durability and follow-up strategy

Recurrence after coil-based therapy remains a practical concern. Liu et al. reported angiographic recurrence during follow-up in a subset of endovascularly treated patients [8]. This supports a follow-up strategy that includes serial vascular imaging. In our case, the six-month magnetic resonance angiography (MRA) showed progression to complete occlusion (Raymond-Roy Class 1) from an immediate postoperative neck remnant (Class 2), suggesting progressive endothelialization of the stent construct. Long-term surveillance is warranted, particularly given the hemodynamic importance of the fetal-type PCoA.

Limitations

The available evidence base for true PCoA aneurysms remains constrained by rarity and publication bias. Comparative conclusions between microsurgery and endovascular approaches are therefore limited, and treatment selection should remain individualized. Additional well-documented cases and longer-term follow-up data would help clarify durability and the role of specific reconstructive strategies in distinct anatomical subtypes.

## Conclusions

We report a rare case of an unruptured true PCoA aneurysm successfully treated with SAC embolization. This case highlights that, in carefully selected patients, endovascular reconstruction can achieve complete aneurysm occlusion while maintaining critical PCoA flow. True PCoA aneurysms have no established management guidelines due to their rarity, but our experience suggests that modern endovascular techniques - employing low-profile stents and adjunctive coiling - represent a safe and effective therapeutic strategy, particularly when open surgery is not feasible. Long-term angiographic follow-up is recommended to ensure treatment durability. Collaborative reporting of such cases will help refine the optimal management of these uncommon aneurysms.
